# A CRISPR endonuclease gene drive reveals distinct mechanisms of inheritance bias

**DOI:** 10.1038/s41467-022-34739-y

**Published:** 2022-11-21

**Authors:** Sebald A. N. Verkuijl, Estela Gonzalez, Ming Li, Joshua X. D. Ang, Nikolay P. Kandul, Michelle A. E. Anderson, Omar S. Akbari, Michael B. Bonsall, Luke Alphey

**Affiliations:** 1 Mathematical Ecology Research Group, Department of Biology, University of Oxford, 11a Mansfield Road, Oxford, OX1 3SZ UK; 2 Arthropod Genetics, The Pirbright Institute, Ash Road, Pirbright, GU24 0NF UK; 3 School of Biological Sciences, Department of Cell and Developmental Biology, University of California San Diego, La Jolla, CA 92093 USA; 4 Present Address: The Department of Biology, University of York, Wentworth Way, York, YO10 5DD UK

**Keywords:** Genetic engineering, CRISPR-Cas systems

## Abstract

CRISPR/Cas gene drives can bias transgene inheritance through different mechanisms. Homing drives are designed to replace a wild-type allele with a copy of a drive element on the homologous chromosome. In *Aedes aegypti*, the sex-determining locus is closely linked to the *white* gene, which was previously used as a target for a homing drive element (*w*^GDe^). Here, through an analysis using this linkage we show that in males inheritance bias of *w*^GDe^ did not occur by homing, rather through increased propagation of the donor drive element. We test the same *w*^GDe^ drive element with transgenes expressing Cas9 with germline regulatory elements *sds3*, *bgcn*, and *nup50*. We only find inheritance bias through homing, even with the identical *nup50*-Cas9 transgene. We propose that DNA repair outcomes may be more context dependent than anticipated and that other previously reported homing drives may, in fact, bias their inheritance through other mechanisms.

## Abstract (web summary)

CRISPR/Cas gene drives can bias transgene inheritance through different mechanisms. Here the authors use gene linkage to show that in males inheritance bias of wGDe did not occur by homing, rather through increased propagation of the donor drive element.

## Introduction

Genetic modification of wild populations through gene drive may be a means of addressing some of the most pressing public health challenges in the world. Gene drive is the ability of a genetic element to bias its own inheritance, allowing it to spread a genetic change throughout a population^1^. There are many examples of natural gene drives that act through different inheritance biasing mechanisms^2^. Some types of gene drive function through the action of enzymes that create sequence-specific DNA breaks (DNA endonucleases), and various context-dependent cellular repair mechanisms exist to resolve DNA breaks^3^. Correspondingly, nuclease-based gene drives can function through different mechanisms including inheritance bias through a copying mechanism (homing drives) and drives that cause the loss of non-drive bearing gametes or offspring (here referred to as meiotic drive).

Generally, in diploid organisms, each parent contributes one chromosome of each homologous pair and each allele has a 50% chance of being passed on to a given progeny (Mendelian inheritance). Synthetic homing and meiotic endonuclease gene drives both rely on selectively creating double-strand DNA breaks on the non-drive-bearing homologue. Through different mechanisms, this results in an inheritance bias of an allele or genomic region and, for meiotic drive, potentially the entire chromosome. Meiotic endonuclease drives lower the inheritance of the competing chromosome within a pair by damaging it, such that gametes carrying the non-drive chromosome are eliminated during gametogenesis or, in some cases, produce non-viable offspring. This includes the disruption of specific essential genes in toxin-antidote meiotic drives^4–6^, or through more structural damage, such as chromosome ’shredder’ meiotic drives^7,8^. Natural sex-linked meiotic drive systems have been reported in *Aedes* and *Culex* mosquitoes^9,10^. Synthetic shredder endonuclease meiotic drives have generally sought to exploit large-scale, potentially repeating sequence differences between chromosome pairs to increase the damage done to the chromosome that does not carry the drive^7,8^.

For most reports of synthetic homing drives, the method of quantifying inheritance bias (phenotypic scoring of progeny carrying a marker gene in the drive allele) cannot differentiate between the underlying inheritance bias mechanism. However, a small subset of reports of homing drives have had marked chromosomes^11–16^, especially pre-CRISPR^17–20^, which may allow homing and meiotic inheritance bias to be differentiated. Through the use of a coincidental chromosomal marker, we observed evidence for meiotic drive in male *A. aegypti* with a homing CRISPR gene drive design reported by Li et al.^12^.

Li et al. tested the inheritance biasing ability of a set of homing split drive systems comprising a guide RNA (gRNA) expressing element inserted into the *white* gene (*w*^GDe^) and one of five secondary site transgene insertions expressing Cas9 under the control of various promoters from genes expressed in the mosquito germline. The *white* gene is tightly linked to the sex-determining region of *A. aegypti* which allows the sex of the progeny to function as a chromosomal marker (donor/recipient) in the progeny of male drive carriers. While three of the Cas9 regulatory regions resulted in drive activity in females, only *nup50* expressing Cas9 resulted in a statistically significant increased inheritance of the drive from male drive parents^12^. We re-analysed the results of Li et al. for *nup50* males taking into account the sex linkage and found that the observed inheritance bias in males seemingly proceeded exclusively through meiotic drive.

We set out to test the hypothesis that the meiotic drive observed with the *nup50* expression pattern is a more general phenomenon and also occurs with other *A. aegypti* gene drives that show activity in males. We repeated the *w*^GDe^ and *nup50*-Cas9 crosses with lines provided by the original authors, and performed crosses with Cas9 expression under the control of putative transcription regulatory regions of two additional *A. aegypti* germline genes. The first, suppressor of defective silencing 3 (*sds3*) has been shown, by dsRNA-induced knockdown in *Anopheles gambiae*, to be necessary for normal development of the ovarian follicles and testes, without other obvious defects^21^. The second, benign gonial cell neoplasm protein (*bgcn*) is involved in the regulation and promotion of gametogenesis in both sexes^22^ and has been described in the context of gene drive in *Drosophila melanogaster* with the I-SceI nuclease^17^.

For each line that expresses Cas9, we report the degree of inheritance bias of the *w*^GDe^ element for both sexes and, in males, the mechanism of inheritance bias. For *sds3*, *bgcn*, and *nup50*-Cas9, we find an increase in recombination events indicative of homing. Furthermore, by scoring somatic eye phenotypes, we also find strong evidence of zygotic/somatic expression, maternal deposition and an effect of the Cas9 carrying grandparent’s sex on *w*^GDe^ inheriting grand-offspring phenotypes.

## Results

### Inheritance of *w*^GDe^ is biased by *bgcn*, *sds3* and *nup50*-Cas9

**Fig. 1:**
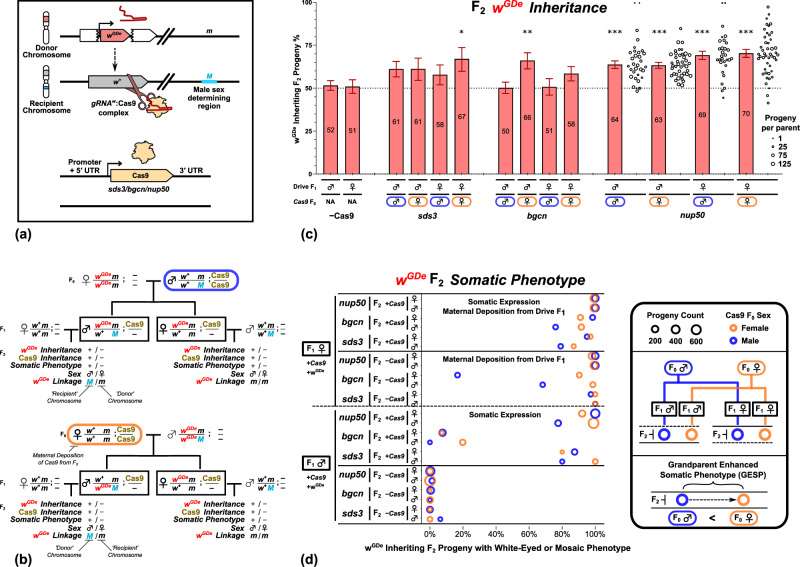
Gene drive element (*w*^GDe^) inheritance and somatic eye phenotype in the progeny of double heterozygote split drive carriers. **a** Illustration of gRNA:Cas9 split drive system. The gene drive element ^GDe^ is inserted into, and disrupts, the *white* gene which is tightly linked to the sex-determining region (*M* or *m*). **b** Breeding schemes for the four crosses per Cas9 expression variant. The solid boxes indicate the F_1_ genotypes that may bias the inheritance of *w*^GDe^ in their germline. The upper family tree shows the double heterozygous F_1_ with paternally contributed Cas9 and maternally contributed *w*^GDe^, *m*-linked in both F_1_ males and females. The bottom family tree shows the double heterozygous F_1_ with paternally contributed *w*^GDe^, *M*-linked in male and *m*-linked in female F_1_s, and maternally contributed Cas9. **c** F_1_ drives parent germline inheritance bias of *w*^GDe^ when combined with a *sds3*, *bgcn* or *nup50*-Cas9 expressing element. The horizontal dotted line indicates the expected Mendelian 50% inheritance. For *nup50*, individual crosses were performed, and each circle represents the percentage of *w*^GDe^ positive progeny from an individual parent. Data are presented as mean values with the Wilson confidence intervals for the binomial proportion calculated for the pooled progeny count, which does not take into account the potential lack of independence due to parent-by-parent batch effects. Stars indicate the *p* value thresholds from two-sided Fisher’s exact tests to the matched drive sex F_1_−Cas9 condition. The *p* values and number of progeny scored are presented in Supplementary Table S9. Source data are provided as a Source Data file. **d** The percentage of *w*^GDe^ inheriting F_2_ progeny, initially of the *w*^+^/*w*^GDe^ genotype, that display a mosaic or total loss of eye pigment phenotype due to disruption of their *w*^+^ allele. The circle size indicates the number of progeny, and circle colour indicates if the Cas9 carrying F_0_ grandparent was male (Blue) or female (Orange). Progeny from F_1_ drive females is indicated with ‘Maternal Deposition’. Progeny that inherited both a *w*^GDe^ allele and Cas9 element are indicated with ‘Somatic Expression’. White phenotype rates for the F_2_ progeny that did not inherit *w*^GDe^ are shown in Supplementary Fig S3.

To assess the degree and, in males, the mechanism of inheritance bias, we bred transgenic *A. aegypti* mosquitoes to create and analyse a split drive arrangement. In this split drive, the *w*^GDe^ allele expresses a gRNA targeting the wildtype *white* gene (*w*^+^) at the site corresponding to where the drive element has been inserted and disrupts its protein coding sequence (Fig. 1a and Supplementary Fig S1). The *white* gene is located on chromosome one, near the dominant acting male determining allele *M* such that males are *M*/*m* and females *m*/*m*.

To generate individuals in which drive can occur, the *w*^GDe^ element is combined with the other component of the split drive, a separate transgene that expresses Cas9 under the control of regulatory sequences from an endogenous germline-specific gene, either *nup50*, *bgcn*, or *sds3*. Individuals carrying a single copy of both the *w*^GDe^ and Cas9 transgenes (double heterozygotes) were generated in two ways: by crossing parental F_0_ female *w*^GDe^ homozygotes to male Cas9 individuals (Fig. 1b Top) or with the reciprocal cross (Fig. 1b Bottom). The double heterozygous offspring (F_1_) were in turn crossed to the Liverpool wild type strain, and their progeny (F_2_) were collected and their fluorescence and phenotype scored (Supplementary Tables S1–S7). For each condition, Fisher’s Exact tests were performed comparing the *w*^GDe^ inheritance rates to those in the absence of any Cas9 element for male (52%, 620/1203) or female (51%, 308/605) parents (Supplementary Table S8). All Cas9 expressing lines were able to bias the inheritance of the *w*^GDe^ element in at least one cross (Supplementary Table S9 and Fig. 1c).

For *sds3*, F_1_ drive females with maternal Cas9 propagated the *w*^GDe^ element to 67% (118/176, *p* value: 0.050^*^) of their progeny (Supplementary Table S1) and for *bgcn*, F_1_ drive males with maternal Cas9 the propagation rate was 66% (257/389, p-value: 0.010^**^) (Supplementary Table S2). For *nup50* (Supplementary Table S3), all four crosses had significantly increased inheritance rates, and to a similar degree as reported to the identical crosses in ref. 12. The *nup50* double heterozygous males passed along the *w*^GDe^ element to 64% (1159/1819, *p* value: 0.001^***^) of their progeny with paternal Cas9 and to 63% (1852/2926, *p* value: <0.001^***^) of their progeny with maternal Cas9. For *nup50* drive females the propagation rate was 69% (952/1377, *p* value: <0.001^***^) for paternal Cas9 and 70% (1055/1501, *p* value: <0.001^***^) for maternal Cas9.

For *nup50*-Cas9, the progeny were collected individually from F_1_ parents (Supplementary Table S4–S7). There was considerable variation between the inheritance rate from different parents carrying the same drive (Fig. 1c), a notable feature that has been reported in other articles on homing drives^11,12,23–25^. Due to this overdispersion, we cannot reliably determine if there is a statistical difference in the inheritance rate between the different Cas9 regulatory elements. However, because this overdispersion is expected only to occur if the drive is functional, our method for determining a difference from the control remains valid, albeit with a potentially inflated false negative rate.

### Eye phenotype reveals the source of nuclease activity

All progeny were evaluated for eye pigment defects that may result from embryonic or later somatic biallelic disruption of the *white* gene by the *w*^GDe^ element and NHEJ mutations. Since the double heterozygote drive-carrying parents were crossed to wildtype individuals, each progeny inherited at least one dominant functional *white* allele from the non-drive parent, and, if the *w*^GDe^ element is not inherited, potentially an additional one from the drive parent. The biallelic loss of function of the *white* gene must therefore occur through deposition into, or somatic expression by, F_2_ individuals. Consistent with this, the progeny of the−Cas9 control crosses did not present with a *white* phenotype (Supplementary Table S8).

For male double heterozygote *sds3*-Cas9 crosses, of the F_2_ progeny (♂ and ♀ pooled) that inherited both the *w*^GDe^ and the Cas9 element, 86% (111/129) presented with a mutant somatic phenotype if the Cas9 carrying F_0_ was male, or 98% (61/62) if the Cas9 carrying F_0_ grandparent was female (F_1_:♂, +Cas9 in Fig. 1d and Supplementary Table S10). For *bgcn*-Cas9 this was 7% (14/196) or 17% (22/129), and for *nup50*-Cas9 this was 95% (586/615) or 98% (924/946). However, if only the *w*^GDe^ element was inherited, no cross had more than 1% of the pooled ♂ and ♀ F_2_ progeny present with a somatic phenotype, presumably resulting from the lack of paternal Cas9 deposition through the sperm (F_1_:♂, −Cas9 in Fig. 1d and Supplementary Table S10). For each cross, this was a significant difference (Supplementary Table S10) indicating somatic expression, without substantial paternal deposition of Cas9/Cas9:gRNA^w^. In contrast to the <1% rate observed in the progeny of F_1_ drive males, the crosses with female double heterozygotes where only the *w*^GDe^ element was inherited, 40% (39/98) of progeny presented with a somatic phenotype if the Cas9 carrying F_0_ was male, while 95% (124/131) if the Cas9 carrying F_0_ grandparent was female. An astounding 99% (75/76) or 100% (61/61) of the *sds3* and 100% (462/462) or 99% (528/535) of the *nup50* progeny presented with somatic phenotypes (F_1_:♀,−Cas9 in Fig. 1d and Supplementary Table S11). This indicates strong maternal deposition of Cas9/Cas9:gRNA^w^. For each cross, this was a significant difference (Supplementary Table S11). Maternal Cas9 deposition without substantial paternal deposition has been reported for many other drive systems^12,14,23–34^.

### Grandparent enhanced somatic phenotype

Surprisingly, in the *w*^GDe^ inheriting progeny, we observed a trend where a higher fraction of the progeny exhibited a somatic phenotype when the Cas9-carrying grandparent was female as opposed to male (F_0_:♂ vs F_0_:♀ in Fig. 1d). Contrasting each male F_0_ Cas9 carrying grandparent cross with the equivalent cross with a female F_0_ Cas9 (each row in Fig. 1d) showed, for female F_0_ Cas9, an average 5.2% (sd:14.4%) percentage point increase in white/mosaic eyed phenotype among + *w*^GDe^ F_2_ progeny. While maternal deposition from a Cas9-carrying grandparent may increase the number of *w*^GDe^ and NHEJ mutated alleles passed along by the F_1_ parental generation to−*w*^GDe^ progeny (Supplementary Fig S3), this should not, in contrast to what we observe (Fig. 1d), influence the phenotype of the progeny that inherit the *w*^GDe^ element. If the *w*^GDe^ element is inherited there is no opportunity to inherit a germline NHEJ mutation that was created due to deposition from the grandparent into the parent. We created a generalised linear model that included Cas9 promoter, F_2_ Cas9 status, F_2_ sex, F_1_ drive parent sex, and F_0_ Cas9 carrying grandparent sex (Supplementary Table S12). The sex of F_0_ Cas9 carrying parent had a significant influence on the fraction of white/mosaic eyed + *w*^GDe^ F_2_ progeny. We termed this phenomenon Grandparent Enhanced Somatic Phenotype (GESP). All other factors were also significant, apart from the sex of the F_2_ progeny.

### Sex of the F_2_ progeny reveals the mechanism of inheritance bias

**Fig. 2:**
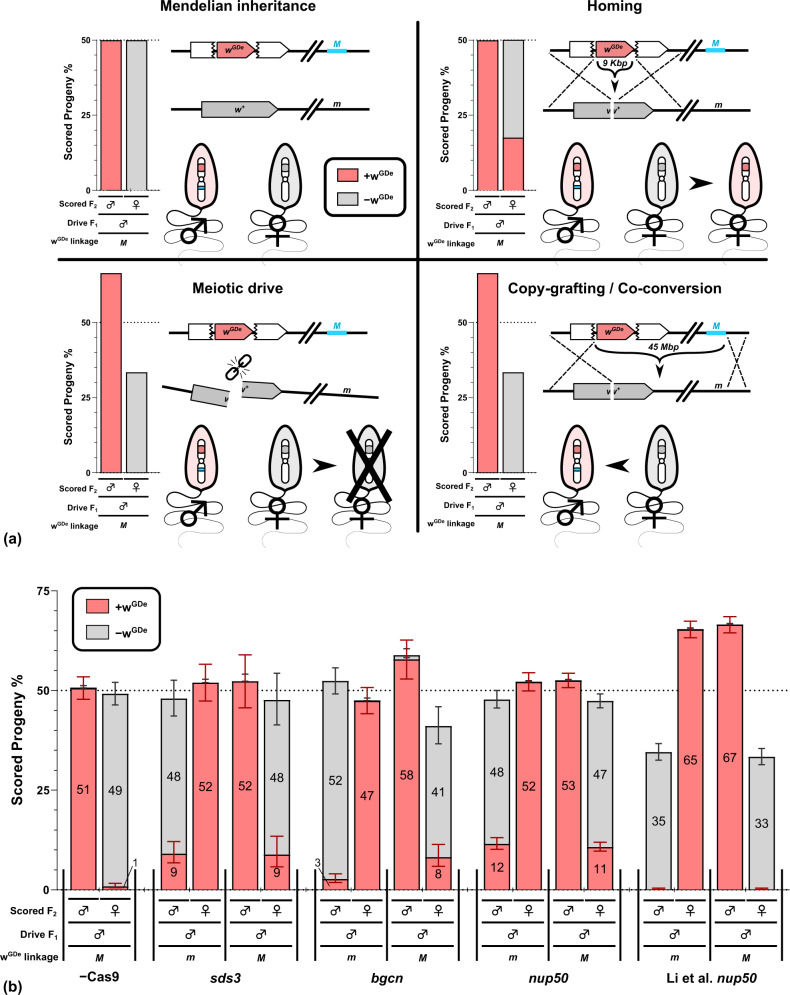
Separating *w*^GDe^ inheritance by F_2_ sex allows different mechanisms of inheritance bias to be distinguished. **a** Illustration of how homing, meiotic drive and copy-grafting/co-conversion are expected to influence the observed sex-linkage of an *M* (shown in Blue) linked *w*^GDe^ element in the progeny of male drive double heterozygous parents. The expected sex-linkage is exactly opposite for an *m* linked *w*^GDe^ element. **b** Parental germline inheritance bias of *w*^GDe^ when combined with no Cas9, *nup50*, *bgcn*, or an *sds3*-Cas9 expressing element. We included the *nup50* results from Li et al. that use the identical *w*^GDe^ and *nup50*-Cas9 line. For each of the three Cas9 regulatory elements, the *w*^GDe^ inheritance from male double heterozygotes is reported in pairs of columns segregated by the sex of the F_2_ progeny. In each case, the first pair of columns are the results for when *w*^GDe^ is *m*-linked (*m*), and the second pair are the results for when *w*^GDe^ is *M*-linked (*M*). Data are presented as mean values with the Wilson confidence intervals for the binomial proportion calculated for the pooled progeny count. The overlaid numbers are the percentage (cumulative within each column) of the indicated F_2_ sex and *w*^GDe^ status among all progeny from that cross. The number of progeny scored is presented in Supplementary Table S3. Source data are provided as a Source Data file.

In *A. aegypti*, the *white* gene is tightly linked to the sex-determining locus. This locus comprises two forms, a dominant male determining allele *M* and a corresponding *m* allele, such that males are *M*/*m* and females *m*/*m*. While the molecular basis of sex determination in this mosquito is not fully understood, *M* is associated with *Nix*, a gene shown to be involved in sex determination^35^. Analogous to an XY chromosome system, male offspring of an *M*/*m* male always carry the paternal *M* allele and female offspring the paternal *m*, with no such distinction between the two *m* alleles of the mother. For the male parent, if the initial linkage of *w*^GDe^ to *m* or *M* is known (determined by the sex of the *wGDe-carrying* grandparent), the sex of the progeny can be used as an indication of whether an observed inheritance bias is due to new recombination events (homing), or increased inheritance of the original drive carrying chromosome (meiotic drive) (Fig. 2a). To this end, we stratified the *w*^GDe^ inheritance by the sex of the F_2_ progeny for each of the double heterozygous male parents (Fig. 2b).

The background recombination rate of *w*^GDe^ and sex in the absence of any Cas9 element was 1.08% (13/1203) (Supplementary Table S8) and was compared by Fisher’s Exact tests to the recombination rate from *w*^GDe^ Cas9 male double heterozygotes (Supplementary Table S13). As reported above, only one cross each of the *sds3* and *bgcn* double heterozygotes showed a significant increase in overall *w*^GDe^ inheritance. However, quantifying conversion with marked chromosomes is much more sensitive than measuring overall *w*^GDe^ inheritance rate.

For the *sds3* double heterozygous males with paternal Cas9 contribution (and therefore in our crosses a *m* linked *w*^GDe^ element) 48% of progeny (216/450) inherited the recipient chromosome as determined by their sex (♂) and 9% of progeny (41/450) were *w*^GDe^ positive males. This allows us to estimate the fraction of recipient chromosomes that were converted by the combined effect of homing and background recombination: 41/216 = 19% (*p* value: <0.001^***^). The same was true for maternally contributed Cas9 where 9% of progeny were *w*^GDe^ females, indicating a homing rate of 19% (19/102 *p*-value: <0.001^***^). For *bgcn* males with paternal or maternal Cas9, we found homing rates of 5% (24/464 *p* value: <0.001^***^) and 20% (32/160 *p* value: <0.001^***^), respectively. This large difference in the rate of homing between crosses with maternal vs. paternal F_0_ Cas9 suggests that for *bgcn* maternally deposited Cas9 may contribute more to homing than autonomously expressed Cas9. Low expression with high maternal deposition by *bgcn*-Cas9 is also consistent with the observed phenotype rates of *white* (Fig. 1d).

For *nup50* double heterozygote males with paternal Cas9 contribution, 24% (210/869 *p* value: <0.001^***^) of the recipient chromosomes were converted by homing. For maternally contributed Cas9 this was 23% (315/1387 *p* value: <0.001^***^) of recipient chromosomes. We also performed this analysis on the *nup50* crosses reported by Li et al. (Supplementary Table S14). Despite a significant increase in inheritance of the *w*^GDe^ element, there was no evidence of an increased recombination rate: 0% (3/690 p-value: 1.0^ns^) for paternal Cas9 and 0% (3/688 *p* value: 1.0^ns^) for maternal Cas9 contribution. Instead, there was a significant bias in favour of the *w*^GDe^ linked sex corresponding to the donor chromosome. For paternally contributed Cas9, 65% (1306/1996 *p* value: <0.001^***^) of progeny were female, >99% of which were *w*^GDe^ positive. For maternally contributed Cas9 67% (1371/2059 *p* value: <0.001^***^) of progeny were male, >99% of which were *w*^GDe^ positive (Supplementary Table S15). This sex bias should not occur through homing, instead, this is consistent with a meiotic drive mechanism where some of the non-*w*^GDe^ chromosomes are lost, or conversion of a very large region encompassing both *w*^GDe^ and the sex-determining region (Fig. 2a). For the crosses performed for this study, including the *nup50* line, no significant difference in sex, and by extension recipient vs donor chromosome inheritance, was detected (Supplementary Table S15). For *bgcn* with maternal F_0_ Cas9, 59% of all F_2_s inherited the donor chromosome (male), but this did not reach our significance threshold due to the relatively low number of progeny scored for this cross.

## Discussion

In this study, we report the efficiency and mechanisms of three CRISPR-Cas9 nuclease gene drives targeting the *white* gene, expanding the set of tools to develop genetic control strategies for the public-health-relevant *A. aegypti* mosquito. In our study, *sds3*, *bgcn* and *nup50* expressed Cas9 each resulted in increased inheritance of the *w*^GDe^ drive element, with the primary mechanism being homing. Additionally, for each promoter, we find evidence of maternal deposition and somatic expression and, unexpectedly, an effect of the Cas9 carrying grandparent’s sex on the grand-offspring phenotypes that we termed GESP. In line with Li et al., we find the *white* locus to be a good drive target, allowing for efficient transmission bias and convenient readout of an easily-scored visible recessive phenotype^12^. In addition, the insertion site allows for effective transgene expression from a sex-linked locus, which may be of particular use for future drives and other genetic control approaches. For the *bgcn* drive in males, the recipient chromosome conversion rate was much higher with maternally contributed Cas9 (19%) compared to paternally contributed Cas9 (5%). These results suggest that, in at least males, the *bgcn* drive may substantially function through maternally contributed Cas9. Homing through Cas9 deposition in the absence of expressed Cas9 (’shadow drive’) has been reported for other drives^11,33,34^, but to our knowledge, not as the primary means of inheritance bias for a drive. We find *nup50* and *sds3*-Cas9 capable of directing transmission bias in females and males, and we did not find that maternal deposition from the Cas9-carrying grandmother negatively influenced the homing rate observed in males. It is important to note that in our crosses only Cas9 could be maternally deposited into the F_1_ double heterozygotes, maternal deposition of Cas9 protein and the gRNA simultaneously may be much less conducive to shadow drive^36^.

For all drives, the almost complete absence of any somatic phenotype in individuals that did not inherit the *w*^GDe^ element (Supplementary Fig S3) could indicate that, while maternal deposition of the Cas9 occurs, the gRNA^w^ or gRNA^w^:Cas9 complex are either not deposited or are rapidly degraded. However, progeny that did not inherit the *w*^GDe^ element instead inherited the (initially) *w*^+^ allele from the double heterozygous parent. For *white* eye phenotypes to occur in these individuals, up to two functional *w*^+^ alleles may need to be disrupted by deposition instead of one; direct comparison of the rates of somatic mutation between offspring that do and do not inherit the *gRNA*^*w*^ transgene are therefore potentially misleading. Furthermore, some non-*w*^GDe^ progeny may have inherited a *white* allele that contained a functional, but cut resistant, NHEJ mutation (type-1 resistant mutation) which would make biallelic disruption impossible.

For the−*w*^GDe^ F_2_ progeny, maternal deposition from the F_0_ grandmother could increase their probability of inheriting a mutated *w* allele. As such, GESP does not apply and only refers to + *w*^GDe^ F_2_ progeny where the sex of the *w*^GDe^ or Cas9-carrying grandparent seemingly influences their propensity to present with a somatic phenotype. Although deposition from an F_0_ grandparent may explain a change in the quantity of *w*^GDe^ alleles passed along by the F_1_ drive parent, it does not appear to explain a change in the phenotype of those F_2_ progeny that inherited a drive element. One possible explanation for GESP may be an increased maternal deposition rate of Cas9:gRNA complexes from increased gRNA expression in *w*^GDe^ homozygous germline cells compared to *w*^GDe^ heterozygous germline cells. Consistent with this, for *bgcn*-Cas9 the *w*^GDe^ homing rate was higher when the Cas9-carrying F_0_ grandparent was female. A similar analysis of a single drive element (containing both Cas9 and a gRNA) found that maternal deposition rates were lower when drive conversion in the maternal germline was less^27^. However, in our split drive system, only the gRNA-expressing element is biased, the Cas9-expressing element remains heterozygous regardless if homing has occurred or not. It may be that different mechanisms, such as genomic imprinting or transgenerational persistence of deposited Cas9 mRNA/protein, contribute to GESP.

For *nup50* the overall inheritance biasing rate and somatic/embryonic drive activity closely match those reported by Li et al.^12^ and underscore its potential utility for systems such as precision-guided SIT^37^. However, an important finding of our work is the propensity of this drive to function through two different mechanisms. The selective inheritance or elimination of a chromosome is generally achieved by creating multiple DNA breaks on the target chromosome^8,38–40^ (e.g. X-shredder) or by disrupting an essential gene^5,6^. Meiotic drive through a single cut in a non-essential gene as found by Li et al. and reported here is noteworthy. An explanation could be the chromosomal location of the induced double-stranded break. A single cut has been shown to be sufficient for inheritance bias through the loss of a chromosome in yeast when it is targeted to a centromere, while nearby sites were not sufficient^41^. Chromosome loss has also been found to be a frequent outcome of allele-specific editing of a pericentromeric site in human embryos^42^. The *white* gene is located relatively near the centromere. However, a centromere effect does not explain the difference in results from this study and that of Li et al., which instead suggests subtle differences in the rearing conditions or background genetics of the mosquito strains may have a significant influence on the underlying mechanisms. Gene drive assessment performed in *D. melanogaster* with different genetic background has revealed differences in drive activity but changes in the underlying mechanism were not investigated^27^. The *nup50*-Cas9 and *w*^GDe^ transgenic lines used in this study are derived from those described in Li et al., but the crosses to assess homing were made to Liverpool (LVP) strains maintained for a long period of time in different insectaries. Mosquito colonies maintained in laboratories can suffer from founder and drift effects, affecting their genetic background and reducing their heterozygosity^43^. Moreover, genetic variability in *A. aegypti* colonies of the same strain but reared in different laboratories has been documented^44^. There may also be methodological factors that could allow the same biological processes to manifest differently (e.g., different screening timings with genotype-dependent mortality rates).

A limitation of our study is that we cannot rule out that the sex bias we report for Li et al. *nup50*-Cas9 is due to copying of the estimated 45Mbp^45,46^ region comprising both the *w*^GDe^ and the sex-determining region (Fig. 2a). However, the large distance between the *w*^GDe^ drive and the *M*/*m* locus leads us to believe that this is unlikely, as co-conversion in similar contexts is generally reported to be on the scale of 100s of base pairs^11,20,47–49^. Furthermore, a substantial fraction of conversion tracts have been reported to be unidirectional in *A. aegypti*^49^. This suggests that even if large-scale co-conversion was favoured, some repair events should still have caused recombination between *w*^GDe^ and the sex-determining locus if co-conversion occurred primarily in the other direction relative to the sex-determination locus. Finally, several studies have reported partial homing events^24,27,31,50–52^. These partial homing events are seemingly due to sequences in the drive element (such as the gRNA gene) having undesired homology to the recipient chromosome (shown for *w*^GDe^ in Supplementary Fig S1) and result in only part of the drive element being copied over. These reports of partial homing are inconsistent with a single DNA break inducing large-scale homing beyond the (immediately) adjacent regions of homology.

There are additional phenomena that can lead to biased inheritance with a sex-linked transgene. In particular, alleles with sex-specific lethal effects may be clustered within the neighbourhood of the sex-determining region in *A. aegypti* and can become linked to a transgene^53^. However, the meiotic drive we report shows a reciprocal sex bias depending on the linkage of the *w*^GDe^ element with the *M* or *m* locus and the use of a split drive system demonstrates that the effects depend on Cas9 activity and are not simply due to the *w*^GDe^ insertion or a linked allele. A more comprehensive analysis of (even more distal) sequence differences between donor and recipient chromosomes after DNA repair may further inform the exact mechanism of inheritance bias. However interpretation of such data must be done with caution, donor chromosome sequences (including the drive element) may incorrectly appear homozygous when NHEJ mutations cause the binding sites of a PCR primer to be blocked on the recipient chromosome. This issue has been raised in several analyses^42,54–56^ and highlights potential pitfalls for identifying homing events with these types of molecular assays. We highlight these cases specifically because we believe such genetic assays are worth perusing, but should be informed by this prior work to reduce the chance of misinterpretation.

To our knowledge, for gene drives designed to function through homing, recipient/donor chromosome markers have been used with non-CRISPR nucleases in *D. melanogaster*^17–19^ and *A. gambiae*^20^ and with CRISPR-Cas9 in *D. melanogaster*^11,15^, *A. aegypti*^12^ and *Mus musculus*^13,16^. There may be additional cases in which a split drive element can coincidentally act as a chromosome marker^14,57^. In *D. melanogaster*, some studies have noted a reduced inheritance of the recipient chromosome, however, these may be attributable to genotype-specific fitness effects instead of DNA damage-induced loss of the recipient chromosome^11,14^. Xu et al. have performed the most extensive investigation of homing drives with marked chromosomes and found a mix of homing and bias through chromosome damage^15^.

In light of our results, re-evaluation of the *A. gambiae* I-SceI gene drive reported by Windbichler et al. may suggest that a meiotic drive effect in homing drive designs is more widespread in mosquitoes^20^. Their drive-carrying line had a small marker (*Not*I restriction site) located ~0.7 kilobases from the I-SceI cut-site on the recipient chromosome, but not on the donor drive chromosome. They reported 86% inheritance of the drive element from heterozygote males. However, drive alleles that included the *Not*I site only accounted for around half the increased drive allele inheritance. The authors attributed this discrepancy to co-conversion, where homing of the drive element also replaced the nearby *Not*I marker. A combined meiotic drive and homing effect would seem to provide an alternative explanation. In the *M. musculus* drive reported by Grunwald et al. the recipient chromosome had a linked coat colour marker that allowed the homing events to be precisely tracked^13^. In females, *vasa*-Cre induced CAG-Cas9 expression resulted in homing rates of 42% (36/86) and 11% (5/47) depending on the Cas9 insertion site. In males, no homing was observed with any drive. However, for the *vasa* drives, males passed along the donor drive chromosome to 63% (45/71) and 54% (49/91) of their progeny, potentially indicating a meiotic drive mechanism in that sex. It should be noted that detecting meiotic drive using this method is less sensitive than detecting homing, and more progeny would need to be scored to have confidence in this trend. Together, these results suggest that a meiotic mechanism in drives intended to function through homing may be more common than currently realised. Distinguishing these mechanisms requires linked markers; for some organisms, this type of in-depth investigation may best be reserved for drives that after initial tests warrant further development.

Our work further expands the Cas9 expression patterns that have been tested in the context of mosquito gene drives. It is notable that the drives with a homing design reported in *Anopheles* mosquitoes *A. gambiae*^23,50,58,59^ and *A. stephensi*^26,30,31^ almost invariably have a dramatically higher conversion rate than those found in *A. aegypti*^12,60^. It is not clear what underlies this difference. However, the fact that the modest conversion rate for *nup50*-Cas9 males remains stable despite a change in the mechanism may limit possible explanations. This stability suggests that the factors that negatively affect the conversion rate in *A. aegypti* are not specific to either homing or meiotic drive. Moreover, it also indicates that the difference in conversion rate observed between mosquito species is probably not due to the species favouring one mechanism over the other. Yet, the difference in mechanism between homing and meiotic drive through gamete destruction has important practical implications: first, the loss of gametes through a meiotic-drive mechanism may negatively affect mating competitiveness by lowering the number of viable gametes, though in some cases gametes may be produced in sufficient excess for this not to be significant. The homing mechanism functions through conversion and should not affect gamete numbers. For the *nup50* meiotic drive reported by Li et al., male *nup50*-Cas9 fecundity was tested and found to not differ from wildtype^12^. Second, on a ’per cut’ basis, meiotic drive is moderately less efficient than homing. When meiotic drive removes a non-drive gamete/embryo, it thereby benefits the remaining gametes/embryos. These may, in addition, to drive carrying gametes, include other wildtype and cut-resistant allele-carrying gametes that were not destroyed. In contrast, homing converts a non-drive gamete to a drive gamete, which does not benefit any of the leftover non-drive gametes making homing more efficient. Third, the linkage between different drive components may vary significantly depending on the mechanisms: for instance, if in a split drive system the Cas9 is located near the gRNA element homing would still only increase the number of gRNA alleles, but not the Cas9 alleles. However, meiotic drive would increase the inheritance of both the gRNA and Cas9 elements. This could theoretically cause a split drive or daisy-chain drive^61^ to spread more than anticipated. Locating each element on separate chromosomes would prevent this, and our data suggest that this may be a wise precaution to increase the predictability of their invasiveness. Although, if anticipated or identified in early-stage field trials, a meiotic drive-induced linkage between elements could also be leveraged, lowering the required release frequencies^62^. Nonetheless, in regards to risk-assessment of rare recombination events, the genomic distance at which two split drive elements become strongly linked is presumably still much more permissive for a meiotic drive mechanism as opposed to a homing mechanism. Last, in the case of Li et al.’s *white* targeting *A. aegypti* drive, its linkage to the sex-determining locus caused an otherwise neutral replacement drive to act, in males, like a sex-biasing suppression drive. This might be desirable for some applications, but surely detrimental if the intended application were different. Most of these concerns apply even if the actual mechanism is co-conversion/copy-grafting of a large chromosome segment as opposed to meiotic drive.

## Methods

### DNA constructs

The sequence and insertion site of the 3xP3-tdTomato carrying gRNA element (Supplementary Fig S1) and *nup50* lines are described in ref. 12 and the *bgcn*-Cas9^63^ and *sds3*-Cas9 constructs were produced by making several alterations to those original plasmids, provided by Omar Akbari^64^. These plasmids contain, within piggyBac terminal sequences, Cas9 expressed by *nup50* followed by a T2A self-cleaving peptide and EGFP and an OpIE2-DsRED cassette. To improve the visibility of the fluorescent marker, this was replaced with PUb-mCherry-SV40 for *bgcn* and *sds3*. To reproduce the germline-specific expression patterns predictoed for these genes, the Cas9:EGFP coding sequence is preceded and followed by the non-coding sequences flanking the endogenous *bgcn* or *sds3* gene’s open reading frame, followed by an additional P10 ${3}^{{\prime} }$UTR. The *bgcn* and *sds3* constructs use a Cas9 that is insect codon optimised^63^. The *nup50* line makes use of a human codon optimised Cas9^64^.

### Mosquito lines

No ethical approval was required for working with the insect lines used in this study. *A. aegypti* Liverpool strain (WT) was a gift from Jarek Krzywinski. The *nup50*-Cas9 and white gRNA expressing element *w*^GDe^ (w^U6b-GDe^) lines were provided by Omar Akbari^12^. The *sds3*-Cas9 line was generated by standard embryo microinjection with a hyper-active *piggy*Bac transposase helper^63^. At Pirbright, the *nup50*-Cas9 line was maintained as a mix of homozygotes and heterozygotes with periodic selective elimination of wildtypes; the *w*^GDe^ element line was provided as homozygous and maintained in our facilities by screening for the white eye phenotype (homozygous knockout of *white*) and the fluorescent marker. Cas9 expressing lines generated at the Pirbright facilities were maintained as heterozygotes, usually by crossing transgenic males to WT females and selecting for the fluorescent marker.

All mosquito lines were reared in an insectary facility under constant conditions of 28 ^∘^C, 65–75% relative humidity and 12:12 light/dark cycle (1h dawn/1h dusk). Larvae were fed ground TetraMin flake fish food (TetraMin) while adults were provided with 10% sucrose solution ad libitum. Defibrinated horse blood (HB034, TCS Bioscience) was provided using a Hemotek membrane feeding system (6W1 system, Hemotek Ltd) covered with Parafilm (HS234526A, Bemis).

### Crosses for homing assessment

Male and female adults, homozygous for *w*^GDe^ were crossed with mosquitoes of the Cas9 lines. Their progeny were screened as late larvae under fluorescence using a Leica MZ165FC microscope. The eye phenotype was also evaluated. Double heterozygous mosquitoes carrying both transgenes were then crossed to WT mosquitoes. Inheritance of the transgenes as well as eye phenotype, was again assessed under a fluorescence microscope. For *nup50*-Cas9, double heterozygotes were individually crossed. For *bgcn*-Cas9 and *sds3*-Cas9 multiple double heterozygotes were crossed simultaneously to WT of the opposite sex. The exact number and phenotype of the progeny of each cross are shown in Supplementary Tables S2–S3. The individual cross data for *nup50*-Cas9 are shown in Supplementary Table S4–S7. In some cases, F_1_ double heterozygotes produced from the same cross presented with a different fluorescent marker or eye pigment phenotypes. In each case, these were noted in the cross tables, and examples of the phenotypes are shown in Supplementary Fig S2.

### Statistical analysis of *w*^GDe^ inheritance bias

For each F_1_ sex, the *w*^GDe^ inheritance rate in the absence of a Cas9 expressing element (Supplementary Table S8) was used as the baseline inheritance. This was 52% (620/1203) for males and 51% (308/605) for females. These rates were used as the expected outcome in a two-sided Fisher’s exact test with the *w*^GDe^ inheritance from F_1_ parents that carried the *w*^GDe^ and one of the Cas9 expressing elements. A significant difference in *w*^GDe^ inheritance is taken as evidence for drive activity. See Supplementary Table S9.

### Statistical analysis of somatic expression and parental deposition

For each Cas9 line, the fraction of mosaic-eyed (ME) or white-eyed (WE) progeny among the F_2_ offspring inheriting *w*^GDe^ but not the Cas9 (+*w*^GDe^; −Cas9) from F_1_ drive males served as a control for the frequency of such phenotypes in the absence of somatic expression or maternal deposition. For somatic expression, the ME/WE fraction of the F_2_ progeny harbouring both the Cas9 and *w*^GDe^ elements from F_1_ drive males was compared to the control cross using a two-sided Fisher’s exact test (Supplementary Table S10). For maternal deposition, the F_2_ progeny harbouring only the *w*^GDe^ element from F_1_ drive females as compared to the control (Supplementary Table S11).

### Statistical analysis of the influence of factors on the fraction of mosaic and white-eyed progeny

A generalised linear model with binomial errors was created that included Cas9 promoter (*sds3*, *bgcn*, *nup50*), F_2_ Cas9 status (+/−), F_2_ sex (♂/♀), F_1_ drive parent sex (♂/♀), and F_0_ Cas9 carrying grandparent sex (♂/♀). The response variable was the proportion of ME and WE progeny among all the F_2_ progeny from that cross and F_2_ sex (48 conditions). The analysis was performed in R version 4.0.2 using the glm function. See Supplementary Table S12.

### Statistical analysis of homing and meiotic drive

For homing, the background recombination rate (calculated from the F_1_ + *w*^GDe^; −Cas9 male cross Supplementary Table S8) is used as the expected outcome in a two-sided Fisher’s exact test. For the control cross (in the absence of possible Cas9 mediated inheritance bias) the *w*^GDe^ allele was provided by the male F_0_ grandparent and therefore *M*-linked in the F_1_ males. In the absence of recombination, all F_2_ males should be *w*^GDe^ positive, and all F_2_ females should be *w*^GDe^ negative. Out of the 1203 progeny scored, we saw 13 (1.08%) recombination events. 2 out of 609 F_2_ males were *w*^GDe^ negative, and 11 out of 581 F_2_ females were *w*^GDe^ positive. For the crosses including a Cas9 element, a statistically significant increase in recombination rate between the recipient/donor chromosome marker and the drive element was taken as evidence of homing (Supplementary Table S13). For meiotic drive, a statistically significant difference in the inheritance of either the recipient or donor chromosome (i.e., F_2_ sex) is taken as evidence for meiotic drive (Supplementary Table S15). The progeny sex ratio is compared to the sex ratio in the absence of a Cas9-expressing element (Supplementary Table S8).

### Reporting summary

Further information on research design is available in the Nature Portfolio Reporting Summary linked to this article.

## Supplementary information

Supplementary Information

Description of Additional Supplementary Files

Reporting Summary

Supplementary Dataset 1

## Data Availability

All the datasets generated during the current study are included in the supplementary information/source data file. The Li et al. data used in this study are available in the supplemental files of the original article 10.7554/eLife.51701.

## Code availability

R version 4.0.2 was used for data analysis. The script for analysis and additional files are available at: https://osf.io/q4cj5/.
