# Supplementary material for: A CRISPR endonuclease gene drive reveals distinct mechanisms of inheritance bias
Source: Nat Commun. 2022 Nov 21;13:7145. doi: 10.1038/s41467-022-34739-y (PMC9681865; doi:10.1038/s41467-022-34739-y)
Supplement: Supplementary file 1 — Supplementary Information [file 41467_2022_34739_MOESM1_ESM.pdf]

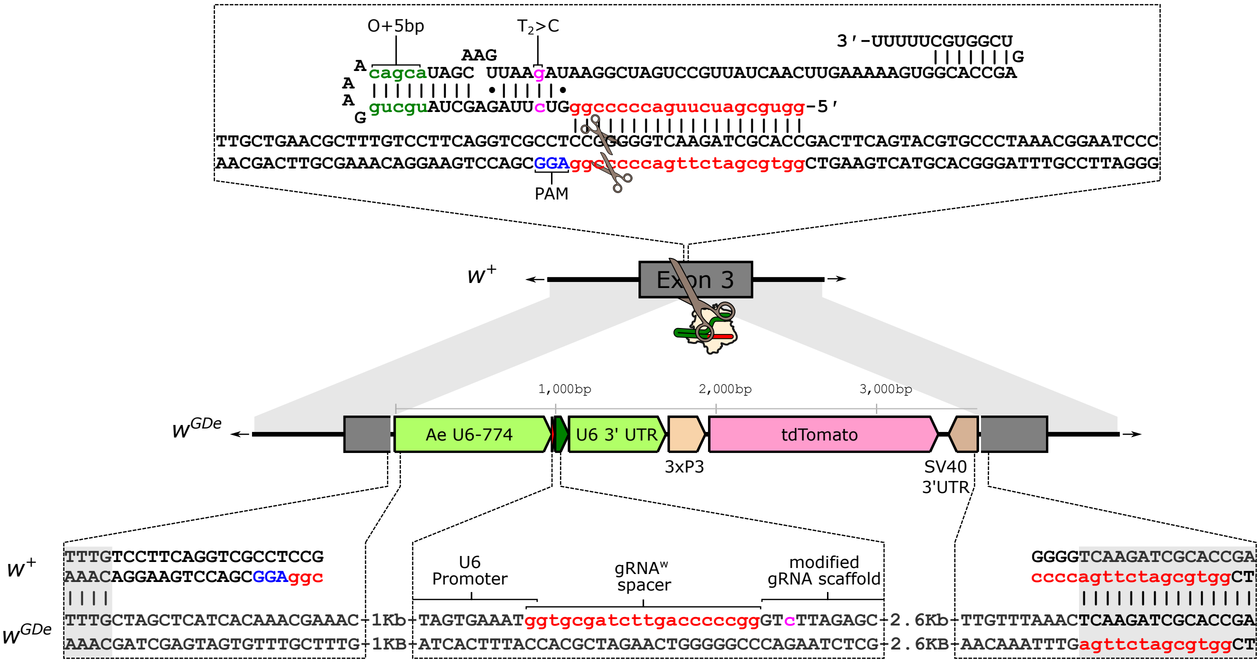

Figure S1. Detailed illustration of the w^{GDe} element and regions of homology with the w^{+} locus after a Cas9:gRNA mediated double-strand DNA break. The gRNA spacer sequence is printed in lowercase red letters as RNA and DNA. The three base pair protospacer adjacent motif (PAM) is printed in Blue letters. Non-standard modifications to the gRNA scaffold sequence are printed in lowercase green (O+5bp) or magenta (T_2>C) letters and are based on Dang et al.[65] and similar modifications have been described in Feng et al.[66]. Regions of homology within the gRNA scaffold, between the gRNA and w^{+}, between the cut w^{+} locus and between the w^{GDe} locus are indicated with vertical lines (|). 22bp of the *white* exon 3 sequence is absent from the sequences flanking the w^{GDe} element. Once w^{+} exon 3 is cut, 18nt and 4nt need to be resected from the free DNA ends for them to become fully homologous to the sequences flanking the w^{GDe} element. The DNA sequence of the gRNA spacer is in the opposite orientation (reverse complement) relative to the same sequences in the flanking homology sequences. This may make it less likely that the gRNA spacer is involved in a partial homing event. The U6 promoter and 3′UTR sequence contain sequences found repeated elsewhere in the genome, in addition to their homology to the endogenous U6b (AAEL017774) gene. Truncations of Pol III promoters suggest that the U6 promoter and 3'UTR sequence used in the w^{GDe} element are likely substantially larger than the minimum sequence required to maintain efficient RNA expression[67].

sds3			$+w^{GDe}$;+Cas9				$+w^{GDe}$;−				−/+Cas9				−/−			
Cross	F_1s crossed (♂x♀)	Drive F_1 phenotype	♀		♂		♀		♂		♀		♂		♀		♂	
			ME/WE	WT	ME/WE	WT	ME/WE	WT	ME/WE	WT	ME/WE	WT	ME/WE	WT	ME/WE	WT	ME/WE	WT
F_1: ♂w^{GDe};*sds3*-Cas9 x ♀LVP<br>F_0: ♂Cas9 x ♀w^{GDe}	10 x 50	Mosaic eyes	91	13	20	5	0	130	1	15	0	0	0	76	0	0	0	99
F_1: ♂LVP x ♀w^{GDe};*sds3*-Cas9<br>F_0: ♂Cas9 x ♀w^{GDe}	10 x 25	Mosaic eyes	38	2	34	9	36	1	39	0	0	22	0	23	0	33	0	38
F_1: ♂w^{GDe};*sds3*-Cas9 x ♀LVP<br>F_0: ♂w^{GDe} x ♀Cas9	10 x 50	Mosaic eyes	4	1	57	0	0	14	0	55	1	40	0	0	0	42	0	0
F_1: ♂LVP x ♀w^{GDe};*sds3*-Cas9<br>F_0: ♂w^{GDe} x ♀Cas9	10 x 21	Mosaic eyes	33	1	20	3	28	0	33	0	0	12	0	16	0	12	1	17

Table S1. F_2 progeny of *sds3*-Cas9 and w^{GDe} double heterozygotes crossed to LVP wildtype. Mosaic eyes (ME), white eyes (WE). Drive F_1 phenotypes are shown in Fig S2.

bgcn			$+w^{GDe}$;+Cas9				$+w^{GDe}$;–				–/+Cas9				–/–			
Cross	F_1s crossed (♂x♀)	Drive F_1 phenotype	♀		♂		♀		♂		♀		♂		♀		♂	
			ME/WE	WT	ME/WE	WT	ME/WE	WT	ME/WE	WT	ME/WE	WT	ME/WE	WT	ME/WE	WT	ME/WE	WT
F_1: ♂w^{GDe};*bgcn*-Cas9 x ♀LVP<br>F_0: ♂Cas9 x ♀w^{GDe}	10 x 50	Dark eyes	11	55	0	1	1	114	0	2	0	0	0	58	0	1	0	101
	10 x 51	Mosaic eyes	3	115	0	11	0	121	0	10	0	0	0	128	0	0	0	153
		Total	14	170	0	12	1	235	0	12	0	0	0	186	0	1	0	254
F_1: ♂LVP x ♀w^{GDe};*bgcn*-Cas9<br>F_0: ♂Cas9 x ♀w^{GDe}	10 x 25	Dark eyes	36	1	32	0	2	37	27	0	0	28	0	41	0	32	0	42
	3 x 7	Mosaic eyes	25	0	6	12	7	8	3	14	0	14	0	12	0	20	0	15
		Total	61	1	38	12	9	45	30	14	0	42	0	53	0	52	0	57
F_1: ♂w^{GDe};*bgcn*-Cas9 x ♀LVP<br>F_0: ♂w^{GDe} x ♀Cas9	5 x 25	Dark eyes	0	8	6	51	0	3	0	60	0	35	0	2	0	40	0	1
	10 x 50	Mosaic eyes	2	18	14	30	0	1	0	64	0	18	1	0	0	35	0	0
		Total	2	26	20	81	0	4	0	124	0	53	1	2	0	75	0	1
F_1: ♂LVP x ♀w^{GDe};*bgcn*-Cas9<br>F_0: ♂w^{GDe} x ♀Cas9	3 x 7	Dark eyes	31	5	43	4	18	6	32	1	0	24	11	17	0	21	0	35
	7 x 20	Mosaic eyes	40	2	44	4	38	0	36	0	0	19	0	34	0	20	0	35
		Total	71	7	87	8	56	6	68	1	0	43	11	51	0	41	0	70

Table S2. F_2 progeny of *bgcn*-Cas9 and w^{GDe} double heterozygotes crossed to LVP wild type. Mosaic eyes (ME), white eyes (WE). The ratio between the amount of dark and mosaic-eyed drive F_1s crossed is roughly proportional to the ratio of those phenotypes generated from the F_0 crosses.

nup50			$+w^{GDe}$;+Cas9				$+w^{GDe}$;–				–/+Cas9				–/–			
Cross	F_1s crossed (♂x♀)	Drive F_1 phenotype	♀		♂		♀		♂		♀		♂		♀		♂	
			ME/WE	WT	ME/WE	WT	ME/WE	WT	ME/WE	WT	ME/WE	WT	ME/WE	WT	ME/WE	WT	ME/WE	WT
F_1: ♂w^{GDe};*nup50*-Cas9 x ♀LVP<br>F_0: ♂Cas9 x ♀w^{GDe}	See Table S4	Mosaic eyes	486	0	100	29	1	462	1	80	0	1	0	306	0	0	0	353
F_1: ♂LVP x ♀w^{GDe};*nup50*-Cas9<br>F_0: ♂Cas9 x ♀w^{GDe}	See Table S5	Mosaic eyes, Brighter	22	0	23	0	15	0	22	0	0	11	0	6	0	10	0	11
		Mosaic eyes, Dimmer	209	0	236	0	207	0	218	0	0	101	0	94	0	91	0	101
		Total	231	0	259	0	222	0	240	0	0	112	0	100	0	101	0	112
F_1: ♂w^{GDe};*nup50*-Cas9 x ♀LVP<br>F_0: ♂w^{GDe} x ♀Cas9	See Table S6	Mosaic eyes, Brighter	106	3	395	8	0	80	0	387	0	243	0	0	0	266	0	0
		Mosaic eyes, Dimmer	57	11	366	0	0	58	0	381	0	281	0	1	0	282	0	1
		Total	163	14	761	8	0	138	0	768	0	524	0	1	0	548	0	1
F_1: ♂LVP x ♀w^{GDe};*nup50*-Cas9<br>F_0: ♂w^{GDe} x ♀Cas9	See Table S7	Mosaic eyes, Brighter	115	2	119	2	109	3	115	1	1	40	0	42	0	34	1	38
		Mosaic eyes, Dimmer	122	0	160	0	144	2	160	1	0	65	0	65	1	78	2	79
		Total	237	2	279	2	253	5	275	2	1	105	0	107	1	112	3	117

Table S3. F_2 progeny of *nup50*-Cas9 and w^{GDe} double heterozygotes crossed to LVP wild type. Mosaic eyes (ME), white eyes (WE). Drive F_1 phenotypes are shown in Fig S2.

Cross	Drive F_1 phenotype	$+w^{GDe}$;+Cas9				$+w^{GDe}$;–				–/+Cas9				–/–			
		♀		♂		♀		♂		♀		♂		♀		♂	
		ME/WE	WT	ME/WE	WT	ME/WE	WT	ME/WE	WT	ME/WE	WT	ME/WE	WT	ME/WE	WT	ME/WE	WT
F_1: ♂w^{GDe};*nup50*-Cas9 x ♀LVP<br>F_0: ♂Cas9 x ♀w^{GDe}	Mosaic	15		5			15		1				8				6
	Mosaic	12		7			11		7				17				19
	Mosaic	29		6	1		23		5				25				15
	Mosaic	34		4			34		4				13				21
	Mosaic	10		1	2		11		1				3				13
	Mosaic	21		8			24		2				14				21
	Mosaic	16		4			8						7				4
	Mosaic	15			1		9		4				12				19
	Mosaic	15		4			19		5				10				14
	Mosaic	1															
	Mosaic	5			2		8		2				7				3
	Mosaic						1										
	Mosaic	18					15		1				10				17
	Mosaic	3					6						3				4
	Mosaic	5		2	2		6		2				4				2
	Mosaic	18		7	2		24		6				11				13
	Mosaic	1											1				0
	Mosaic	19		4	2		18		8				6				8
	Mosaic	12					8						6				6
	Mosaic	19		4			15		9				2				7
	Mosaic	22		1			33		2				8				20
	Mosaic	23		5			11		5				9				9
	Mosaic	7		1			3						5				3
	Mosaic	10		1	2	1	15	1	3				8				9
	Mosaic	32		9			21		1				9				20
	Mosaic	16		2	3		23		3				15				12
	Mosaic	21		5	2		17						25				13
	Mosaic	21		4			11		3		1		7				8
	Mosaic	5		6			14						12				13
	Mosaic	15		1	3		9						11				8
	Mosaic	24		5	3		21		2				13				23
	Mosaic	22		4	4		29		4				25				23
	Total	486	0	100	29	1	462	1	80	0	1	0	306	0	0	0	353

Table S4. F_2 progeny of individual male *nup50*-Cas9 and w^{GDe} double heterozygotes crossed to LVP wild type. *nup50*-Cas9 from paternal F_0. Mosaic eyes (ME), white eyes (WE). Drive F_1 phenotypes are shown in Fig S2.

Cross	Drive F_1 phenotype	$+w^{GDe}$;+Cas9				$+w^{GDe}$;–				–/+Cas9				–/–			
		♀		♂		♀		♂		♀		♂		♀		♂	
		ME/WE	WT	ME/WE	WT	ME/WE	WT	ME/WE	WT	ME/WE	WT	ME/WE	WT	ME/WE	WT	ME/WE	WT
F_1: ♂LVP x ♀w^{GDe};*nup50*-Cas9 F_0: ♂Cas9 x ♀w^{GDe}	Mosaic, Dimmer	6		5		8		2			3		4		2		2
	Mosaic, Dimmer	10		9		10		7			1		3		3		4
	Mosaic, Dimmer	7		6		7		6			4				4		5
	Mosaic, Dimmer	20		12		0		11					5		3		4
	Mosaic, Dimmer	7		12		8		15			5		4		5		5
	Mosaic, Dimmer	5		15		9		20			5				9		4
	Mosaic, Dimmer	12		14		11		10			8		5		4		8
	Mosaic, Dimmer	3				1									1		
	Mosaic, Dimmer	3		4		4		6			8		3		4		3
	Mosaic, Dimmer	7		6		2		6			4		4		6		5
	Mosaic, Dimmer	9		6		13		7			2		3		4		8
	Mosaic, Dimmer					2											
	Mosaic, Dimmer	5		9		17		13			10		5		3		4
	Mosaic, Dimmer	17		16		9		14			5		4		3		4
	Mosaic, Dimmer	7		17		5		12			3		5		5		5
	Mosaic, Dimmer	4		8		13		2			2		7		4		7
	Mosaic, Dimmer	3		11		3		2			6		4		1		2
	Mosaic, Dimmer	13		5		10		5			5		2		6		3
	Mosaic, Dimmer	19		16		7		8			6		6		3		4
	Mosaic, Dimmer	4		4		8		6			6		6		5		5
	Mosaic, Dimmer	6		5		3		5			2		1		6		2
	Mosaic, Dimmer	12		15		18		14			12		7		3		2
	Mosaic, Dimmer	9		15		16		18			2		7		2		3
	Mosaic, Dimmer	9		10		8		11					2				4
	Mosaic, Dimmer	12		16		15		18			2		7		5		8
	Mosaic, Brighter	5		7		4		6			1		3		3		4
	Mosaic, Brighter	6		5		4		1			2				3		3
	Mosaic, Brighter	8		11		5		14			6		2		3		3
	Mosaic, Brighter	2															
	Mosaic, Brighter	1				2		1			2		1		1		1
	Sum	231	0	259	0	222	0	240	0	0	112	0	100	0	101	0	112

Table S5. F_2 progeny of individual female *nup50*-Cas9 and w^{GDe} double heterozygotes crossed to LVP wild type. *nup50*-Cas9 from paternal F_0. Mosaic eyes (ME), white eyes (WE). Drive F_1 phenotypes are shown in Fig S2.

Cross	Drive F_1 phenotype	$+w^{GDe}$;+Cas9				$+w^{GDe}$;–				–/+Cas9				–/–			
		♀		♂		♀		♂		♀		♂		♀		♂	
		ME/WE	WT	ME/WE	WT	ME/WE	WT	ME/WE	WT	ME/WE	WT	ME/WE	WT	ME/WE	WT	ME/WE	WT
F_1: ♂w^{GDe};*nup50*-Cas9 x ♀LVP F_0: ♂w^{GDe} x ♀Cas9	Mosaic, Brighter	7		26			6		21		13				15		
	Mosaic, Brighter	4		17			1		15		9				14		
	Mosaic, Brighter	3		14			5		12		2				4		
	Mosaic, Brighter	7		35			10		25		11				12		
	Mosaic, Brighter	6		21			4		26		11				14		
	Mosaic, Brighter	4		26			3		22		24				17		
	Mosaic, Brighter	9		13			3		27		6				16		
	Mosaic, Brighter	4		11			6		6		4				12		
	Mosaic, Brighter	2		10	1		3		10		7				14		
	Mosaic, Brighter		3	17			1		14		11				15		
	Mosaic, Brighter	7		21			5		17		6				9		
	Mosaic, Brighter	11		12			4		14		9				7		
	Mosaic, Brighter	9		25			5		26		14				14		
	Mosaic, Brighter	6		33			7		26		32				25		
	Mosaic, Brighter	4		17			3		10		11				7		
	Mosaic, Brighter	6		12			2		15		11				10		
	Mosaic, Brighter	1		17			3		22		7				12		
	Mosaic, Brighter	2		12	7		4		17		15				7		
	Mosaic, Brighter	5		18					19		19				9		
	Mosaic, Brighter	1		17			2		16		6				15		
	Mosaic, Brighter	8		21			3		27		15				18		
	Mosaic, Dimmer			25			1		28		23				15		
	Mosaic, Dimmer	3		26					31		30				26		
	Mosaic, Dimmer	3		27			3		20		15				23		
	Mosaic, Dimmer	2		17			3		24		24				18		
	Mosaic, Dimmer	4		23			2		22		13				13		
	Mosaic, Dimmer	2		20			3		15		10		1		11		
	Mosaic, Dimmer	3		16			1		25		18				14		
	Mosaic, Dimmer	16		29			13		32		21				17		
	Mosaic, Dimmer		2	7					7		2				4		
	Mosaic, Dimmer			22			4		13		14				19		
	Mosaic, Dimmer	3		14			3		16		16				14		
	Mosaic, Dimmer	1		11					12		4				5		
	Mosaic, Dimmer	1		12			1		20		9				7		
	Mosaic, Dimmer		4	12					8		3				10		
	Mosaic, Dimmer	3		30			5		28		17				22		
	Mosaic, Dimmer	6		21			8		31		27				22		
	Mosaic, Dimmer	1	2	13			4		12		9				14		
	Mosaic, Dimmer	5	1	9			4		9		8				8		
	Mosaic, Dimmer	3		18			2		18		14				12		1
	Mosaic, Dimmer	1	2	14			1		10		4				8		
	Sum	163	14	761	8	0	138	0	768	0	524	0	1	0	548	0	1

Table S6. F_2 progeny of individual male *nup50*-Cas9 and w^{GDe} double heterozygotes crossed to LVP wild type. *nup50*-Cas9 from maternal F_0. Mosaic eyes (ME), white eyes (WE). Drive F_1 phenotypes are shown in Fig S2.

Cross	Drive F_1 phenotype	$+w^{GDe}$;+Cas9				$+w^{GDe}$;−				−/+Cas9				−/−			
		♀		♂		♀		♂		♀		♂		♀		♂	
		ME/WE	WT	ME/WE	WT	ME/WE	WT	ME/WE	WT	ME/WE	WT	ME/WE	WT	ME/WE	WT	ME/WE	WT
F_1: ♂LVP x ♀w^{GDe};*nup50*-Cas9<br>F_0: ♂w^{GDe} x ♀Cas9	Mosaic, Brighter	0				1							1				
	Mosaic, Brighter	2		3		9		7			3		3		2		1
	Mosaic, Brighter	3		4		5		2			0						1
	Mosaic, Brighter	10	1	13		6	1	11	1	1	3		1				1
	Mosaic, Brighter	12		9		6		8			2		3		5		5
	Mosaic, Brighter	1		6		6		6			2		2		4		1
	Mosaic, Brighter	16	1	13		6	2	4			1						
	Mosaic, Brighter	2		2		3		3			0				1		
	Mosaic, Brighter	4		6		5		5			9		2		4		3
	Mosaic, Brighter	4		8	2	6		4			2		3				
	Mosaic, Brighter	5		5		3		7					3		3		3
	Mosaic, Brighter	3		9		5		3			3		1		2		1
	Mosaic, Brighter	18		16		10		16			5		3		6		8
	Mosaic, Brighter	4		2		3		2			1		1				1
	Mosaic, Brighter	4		5		10		5					3			1	2
	Mosaic, Brighter	6		3		5		3					3		1		3
	Mosaic, Brighter	2				1		3					1				3
	Mosaic, Brighter	7		4		5		10			2		1		2		1
	Mosaic, Brighter	3		3		4		5			3		6		1		2
	Mosaic, Brighter	5		1		5		3					2				1
	Mosaic, Brighter	1		3		2		7					2		1		1
	Mosaic, Brighter	3		4		3		1			4		1		2		
	Mosaic, Dimmer	9		8		9		8			2		5		6		3
	Mosaic, Dimmer	2		4		7		1			3		4		4		
	Mosaic, Dimmer	7		4		5		7			4		3	1	3		5
	Mosaic, Dimmer	6		10		7		3			2		3		2		4
	Mosaic, Dimmer	10		10		3		11			3		2		3		8
	Mosaic, Dimmer	4		6		6		12					2		1		1
	Mosaic, Dimmer	6		6		1		8			5		4		2		4
	Mosaic, Dimmer	6		5		6		9			5		1		2		2
	Mosaic, Dimmer	8		7		12		3			4		3		2	1	1
	Mosaic, Dimmer	2		2		5		7			1		4		3		
	Mosaic, Dimmer	13		17		14		9			4		2		3		10
	Mosaic, Dimmer	5		8		9		10			4		2		4		4
	Mosaic, Dimmer	6		10			2	3	1							1	3
	Mosaic, Dimmer	2		5				6									2
	Mosaic, Dimmer	3		5		4					1		1		2		3
	Mosaic, Dimmer	8		8		10		8			5		3		7		9
	Mosaic, Dimmer	5		5		2		12			3		2		3		3
	Mosaic, Dimmer	3		3		4		3			2		2		3		3
	Mosaic, Dimmer			2		3		6			2		1		1		2
	Mosaic, Dimmer	4		6		9		12			1		3		5		5
	Mosaic, Dimmer	1		6		8		1			5		5		9		
	Mosaic, Dimmer	2		2		2		6			3		7		3		4
	Mosaic, Dimmer	5		8		9		9			2		4		6		3
	Mosaic, Dimmer	5		13		9		6			4		2		4		
	Sum	237	2	279	2	253	5	275	2	1	105	0	107	1	112	3	117

Table S7. F_2 progeny of individual female *nup50*-Cas9 and w^{GDe} double heterozygotes crossed to LVP wildtype. *nup50*-Cas9 from maternal F_0. Mosaic eyes (ME), white eyes (WE). Drive F_1 phenotypes are shown in Fig S2.

Cas9	Drive F_1 phenotype	$+w^{GDe}$;+Cas9				$+w^{GDe}$;−				−/+Cas9				−/−			
Cross		♀		♂		♀		♂		♀		♂		♀		♂	
		ME/WE	WT	ME/WE	WT	ME/WE	WT	ME/WE	WT	ME/WE	WT	ME/WE	WT	ME/WE	WT	ME/WE	WT
F_1: ♂w^{GDe} x ♀LVP<br>F_0: ♂w^{GDe} x ♀ LVP	Dark eyes	0	0	0	0	0	11	0	609	0	0	0	0	0	581	0	2
F_1: ♂LVP x ♀w^{GDe}<br>F_0: ♂w^{GDe} x ♀ LVP	Dark eyes	0	0	0	0	0	145	0	163	0	0	0	0	0	147	0	150

Table S8. F_2 progeny of w^{GDe} heterozygotes (no Cas9) crossed to LVP wild type. Mosaic eyes (ME), white eyes (WE)

Cas9	Cas9 F_0	Drive F_1	$+w^{GDe}$ F_2	Total F_2	%	p-value	Threshold	Odds ratio
-Cas9	N/A	♂	620	1203	51.5%	1.00E+00	ns	1
-Cas9	N/A	♀	308	605	50.9%	1.00E+00	ns	1
sds3	♂	♂	275	450	61.1%	6.58E-02	ns	1.2
sds3	♀	♂	131	214	61.2%	1.57E-01	ns	1.2
sds3	♂	♀	159	275	57.8%	2.98E-01	ns	1.1
sds3	♀	♀	118	176	67.0%	4.95E-02	*	1.3
bgcn	♂	♂	444	885	50.2%	7.32E-01	ns	1.0
bgcn	♀	♂	257	389	66.1%	9.75E-03	**	1.3
bgcn	♂	♀	210	414	50.7%	1.00E+00	ns	1.0
bgcn	♀	♀	304	520	58.5%	1.75E-01	ns	1.1
nup50	♂	♂	1159	1819	63.7%	6.32E-04	***	1.2
nup50	♀	♂	1852	2926	63.3%	3.76E-04	***	1.2
nup50	♂	♀	952	1377	69.1%	1.67E-04	***	1.4
nup50	♀	♀	1055	1501	70.3%	6.61E-05	***	1.4

Table S9. Overall w^{GDe} inheritance bias. in each case, a two-sided Fisher's exact test is performed using the $-$Cas9 condition with the matching drive F_1 sex. Significance thresholds: * for $\leq$0.05, ** for $\leq$0.01, *** for $\leq$0.001.

Cas9	Cas9 F_0	Drive F_1	F_2 Cas9	F_2 w^{GDe}	ME/WE	Total	%	Control Difference	Ctrl ME/WE	Ctrl Total	Ctrl %	p-value	Threshold	Odds ratio
sds3	♂	♂	Yes	Yes	111	129	86.0%	Cas9 Positive Vs Negative	1	146	0.7%	1.58E-27	***	124.6
sds3	♀	♂	Yes	Yes	61	62	98.4%	Cas9 Positive Vs Negative	0	69	0.0%	1.65E-15	***	Inf
bgcn	♂	♂	Yes	Yes	14	196	7.1%	Cas9 Positive Vs Negative	1	248	0.4%	2.07E-04	***	17.6
bgcn	♀	♂	Yes	Yes	22	129	17.1%	Cas9 Positive Vs Negative	0	128	0.0%	1.04E-06	***	Inf
nup50	♂	♂	Yes	Yes	586	615	95.3%	Cas9 Positive Vs Negative	2	544	0.4%	1.45E-118	***	258.7
nup50	♀	♂	Yes	Yes	924	946	97.7%	Cas9 Positive Vs Negative	0	906	0.0%	9.62E-205	***	Inf

Table S10. Two-sided Fisher's exact test for somatic expression. Mosaic eyes (ME), white eyes (WE). Significance thresholds: * for $\leq$0.05, ** for $\leq$0.01, *** for $\leq$0.001.

Cas9	Cas9 F_0	Drive F_1	F_2 Cas9	F_2 w^{GDe}	ME/WE	Total	%	Control Difference	Ctrl ME/WE	Ctrl Total	Ctrl %	p-value	Threshold	Odds ratio
sds3	♂	♀	No	Yes	75	76	98.7%	Drive F_1 ♀ vs ♂	1	146	0.7%	1.80E-26	***	141.9
sds3	♀	♀	No	Yes	61	61	100.0%	Drive F_1 ♀ vs ♂	0	69	0.0%	7.36E-16	***	Inf
bgcn	♂	♀	No	Yes	39	98	39.8%	Drive F_1 ♀ vs ♂	1	248	0.4%	1.49E-18	***	97.9
bgcn	♀	♀	No	Yes	124	131	94.7%	Drive F_1 ♀ vs ♂	0	128	0.0%	1.61E-28	***	Inf
nup50	♂	♀	No	Yes	462	462	100.0%	Drive F_1 ♀ vs ♂	2	544	0.4%	2.29E-115	***	271.3
nup50	♀	♀	No	Yes	528	535	98.7%	Drive F_1 ♀ vs ♂	0	906	0.0%	8.43E-178	***	Inf

Table S11. Two-sided Fisher's exact test for maternal deposition. Mosaic eyes (ME), white eyes (WE). Significance thresholds: * for $\leq$0.05, ** for $\leq$0.01, *** for $\leq$0.001.

Predictors	Estimate	S.E.	z-value	p-value	Threshold
bgcn-Cas9	-3.5012	0.2201	-15.909	<2e-16	***
nup50-Cas9	0.9341	0.1995	4.683	2.83E-06	***
+Cas9	6.2214	0.1759	35.365	<2e-16	***
Cas9 F_0 female	0.8699	0.1442	6.033	1.61E-09	***
gRNA:Cas9 F_1 female	8.0606	0.2280	35.351	<2e-16	***
Scored F_2 female	0.2328	0.1397	1.667	0.0955	ns
Constant	-5.0034	0.2673	-18.721	<2e-16	***

Table S12. The results of the binomial generalised linear model applied to mosaic eyed (ME) or white-eyed (WE) phenotype fraction among all $+w^{GDe}$ F_2 progeny in Table S1-S3. No interaction terms were specified. The *sds3*-Cas9, -Cas9, Cas9 F_0 male, gRNA:Cas9 F_1 male, F_2 male cross serves as the reference result. Significance thresholds: * for $\leq$0.05, ** for $\leq$0.01, *** for $\leq$0.001.

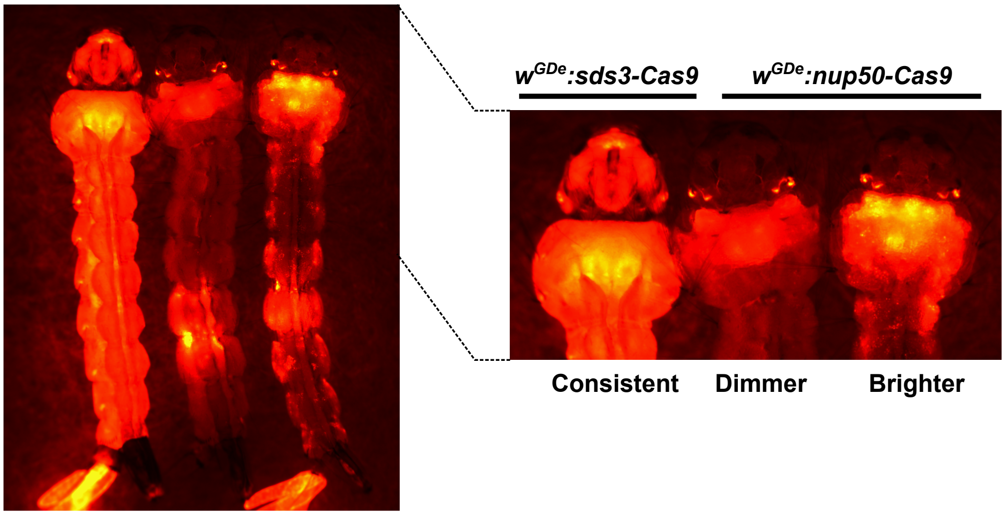

(a)

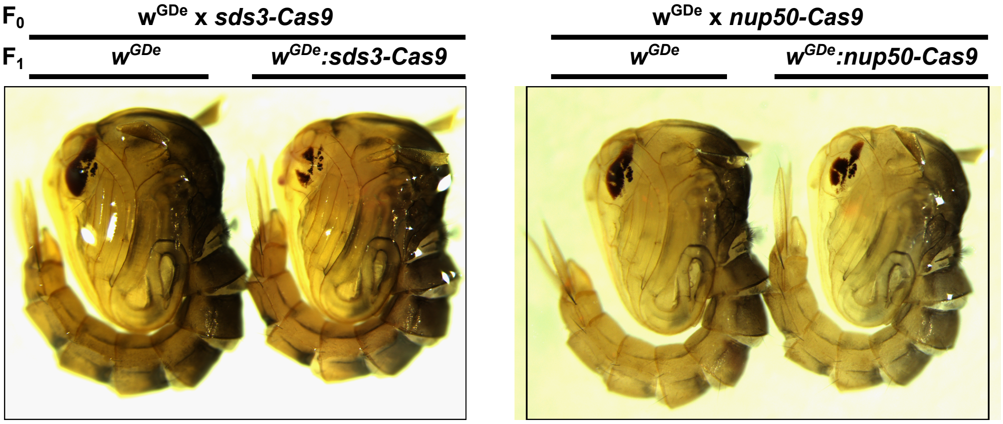

(b)

Figure S2. Fluorescent marker phenotype and *white* gene eye phenotype noted among the F_1s. a 'Dimmer' and 'Brighter' OpIE2-DsRED phenotype noted in the *nup50*-Cas9 line. The *sds3* and *bgcn*-Cas9 used PUb-mCherry-SV40 and had a consistent phenotype. b Examples of the F_1 dark-eyed (WT) and mosaic eyed phenotype.

Cas9	Drive F_1	Cas9 F_0	Positives	Total Events	%	p-value	Threshold	Odds ratio
-Cas9	♂	N/A	11	592	1.9%	1.95E-01	ns	1.7
sds3	♂	♂	41	216	19.0%	6.79E-22	***	17.5
sds3	♂	♀	19	102	18.6%	4.93E-13	***	17.2
bgcn	♂	♂	24	464	5.2%	3.99E-06	***	4.8
bgcn	♂	♀	32	160	20.0%	2.89E-19	***	18.4
nup50	♂	♂	210	869	24.2%	3.50E-57	***	22.3
nup50	♂	♀	315	1387	22.7%	1.02E-61	***	21.0
Li et al. *nup50*	♂	♂	3	690	0.4%	1.93E-01	ns	0.4
Li et al. *nup50*	♂	♀	3	688	0.4%	1.94E-01	ns	0.4

Table S13. Homing significance test based on the recombination of the $+w^{GDe}$ allele and sex-determining region. The total events are all female F_2s for the ♀ Cas9 F_0 (and therefore ♂ w^{GDe} F_0) crosses. Positives are $+w^{GDe}$ female F_2s which should only occur through recombination. For ♂ Cas9 F_0 crosses male F_2s are considered. In each case, a two-sided Fisher's exact test is performed with 13/1203 as the expected outcome (this includes recombination of the *white* allele in the -Cas9 cross). Significance thresholds: * for $\leq$0.05, ** for $\leq$0.01, *** for $\leq$0.001.

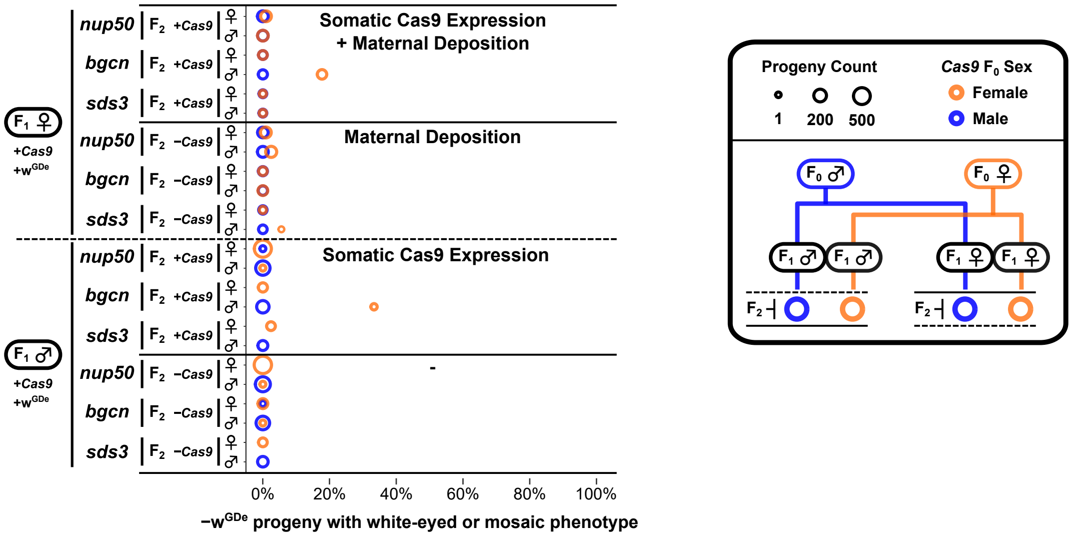

(a)

Figure S3. Somatic eye phenotype in the $-w^{\mathrm{GDe}}$ progeny of double heterozygote split drive carriers. The percentage of $-w^{\mathrm{GDe}}$ progeny that display a mosaic or total loss of eye pigment phenotype. F_2 progeny are segregated by the drive carrying F_1's sex (♂, ♀), the F_2's Cas9 transgene inheritance (−Cas9, +Cas9), the Cas9 regulatory sequences (*sds3*, *bgcn*, *nup50*), and the F_2's sex (♂, ♀). The circle size indicates the number of progeny that make up that group, and circle colour indicates if the Cas9 carrying F_0 grandparent was male (Blue) or female (Orange). The set of progeny that came from drive F_1 carrying females are indicated with 'Maternal Deposition'. The set of progeny that inherited a Cas9 element are indicated with 'Somatic Cas9 Expression'. Due to the sex-linkage of the w^{GDe} element from F_1 drive males the number of F_2 progeny are unequally distributed among the groups for each sex. This sex-bias is more pronounced for the $-w^{\mathrm{GDe}}$ progeny than for the $+w^{\mathrm{GDe}}$ progeny due to the the effects of homing. Circles are absent if no progeny matched that grouping. Within matched crosses (each row), differences in the white phenotype rate corresponding to the Cas9 carrying F_0's sex cannot be solely attributed to a grandparent enhanced somatic phenotype for $-w^{\mathrm{GDe}}$ progeny. White phenotype rates for $+w^{\mathrm{GDe}}$ progeny are shown in Fig 1d. Note that inheritance of the w^{GDe} element also prevents the potential inheritance from the drive parent of an undamaged wild-type *white* gene or a *white* gene mutation that retains its function (r1) in pigment production. As such, any difference in white phenotype rates between $+w^{\mathrm{GDe}}$ and $-w^{\mathrm{GDe}}$ progeny cannot be solely attributed to gRNA expression from w^{GDe} in the F_2 progeny.

Li et al. *nup50*	$+w^{\mathrm{GDe}}$				$-w^{\mathrm{GDe}}$			
Cross	♀		♂		♀		♂	
	ME/WE	WT	ME/WE	WT	ME/WE	WT	ME/WE	WT
F_1: ♂w^{GDe};*nup50*-Cas9 x ♀LVP<br>F_0: ♂Cas9 x ♀w^{GDe}	650	654	0	3	0	2	0	687
F_1: ♂LVP x ♀w^{GDe};*nup50*-Cas9<br>F_0: ♂Cas9 x ♀w^{GDe}	757	0	790	0	0	162	0	175
F_1: ♂w^{GDe};*nup50*-Cas9 x ♀LVP<br>F_0: ♂w^{GDe} x ♀Cas9	0	3	701	669	0	685	0	1
F_1: ♂LVP x ♀w^{GDe};*nup50*-Cas9<br>F_0: ♂w^{GDe} x ♀Cas9	743	0	763	0	3	175	1	185

Table S14. F_2 progeny of *nup50*-Cas9 and w^{GDe} double heterozygotes crossed to wild type taken from Li et al. Mosaic eyes (ME), white eyes (WE). Data are, in row order, from Li et al. supplemental files 4f, 4e, 4d, and 4c.

Cas9	drive F_1	Cas9 F_0	♂ F_2s	♂+♀ F_2s	%	p-value	Threshold	Odds ratio
-Cas9	♂	N/A	611	1203	50.8%	1.00E+00	ns	1.00
sds3	♂	♂	216	450	48.0%	5.65E-01	ns	0.95
sds3	♂	♀	112	214	52.3%	8.49E-01	ns	1.03
bgcn	♂	♂	464	885	52.4%	6.77E-01	ns	1.03
bgcn	♂	♀	229	389	58.9%	1.29E-01	ns	1.16
nup50	♂	♂	869	1819	47.8%	3.48E-01	ns	0.94
nup50	♂	♀	1539	2926	52.6%	5.57E-01	ns	1.04
Li et al. *nup50*	♂	♂	690	1996	34.6%	7.91E-09	***	0.68
Li et al. *nup50*	♂	♀	1371	2059	66.6%	8.10E-06	***	1.31

Table S15. Meiotic drive significance test based on sex bias in F_2 progeny. In each case, a two-sided Fisher's exact test is performed with 611/1203 as the expected outcome. Significance thresholds: * for ≤ 0.05, ** for ≤ 0.01, *** for ≤ 0.001.
