# Supplementary material for: A CRISPR endonuclease gene drive reveals distinct mechanisms of inheritance bias
Source: Nat Commun. 2022 Nov 21;13:7145. doi: 10.1038/s41467-022-34739-y (PMC9681865; doi:10.1038/s41467-022-34739-y)
Supplement: Supplementary file 2 — Description of Additional Supplementary Files [file 41467_2022_34739_MOESM2_ESM.pdf]

Title: Supplementary Dataset 1.
Description: This file contains tables with the scored F2 progeny used to produce the figures and analyses in this study. These data are also shown in the supplemental file with additional information in the table captions.
