# Supplementary material for: A CRISPR endonuclease gene drive reveals distinct mechanisms of inheritance bias
Source: Nat Commun. 2022 Nov 21;13:7145. doi: 10.1038/s41467-022-34739-y (PMC9681865; doi:10.1038/s41467-022-34739-y)
Supplement: Supplementary file 3 — Reporting Summary [file 41467_2022_34739_MOESM3_ESM.pdf]

nature portfolio

Corresponding author(s): Luke Alphey

Last updated by author(s): Nov 3, 2022

Reporting Summary

Nature Portfolio wishes to improve the reproducibility of the work that we publish. This form provides structure for consistency and transparency in reporting. For further information on Nature Portfolio policies, see our Editorial Policies and the Editorial Policy Checklist.

Statistics

For all statistical analyses, confirm that the following items are present in the figure legend, table legend, main text, or Methods section.

n/a	Confirmed	
[ ]	[x]	The exact sample size (n) for each experimental group/condition, given as a discrete number and unit of measurement
[ ]	[x]	A statement on whether measurements were taken from distinct samples or whether the same sample was measured repeatedly
[ ]	[x]	The statistical test(s) used AND whether they are one- or two-sided<br>*Only common tests should be described solely by name; describe more complex techniques in the Methods section.*
[ ]	[x]	A description of all covariates tested
[x]	[ ]	A description of any assumptions or corrections, such as tests of normality and adjustment for multiple comparisons
[ ]	[x]	A full description of the statistical parameters including central tendency (e.g. means) or other basic estimates (e.g. regression coefficient) AND variation (e.g. standard deviation) or associated estimates of uncertainty (e.g. confidence intervals)
[ ]	[x]	For null hypothesis testing, the test statistic (e.g. F, t, r) with confidence intervals, effect sizes, degrees of freedom and P value noted<br>*Give P values as exact values whenever suitable.*
[x]	[ ]	For Bayesian analysis, information on the choice of priors and Markov chain Monte Carlo settings
[x]	[ ]	For hierarchical and complex designs, identification of the appropriate level for tests and full reporting of outcomes
[x]	[ ]	Estimates of effect sizes (e.g. Cohen's d, Pearson's r), indicating how they were calculated

Our web collection on statistics for biologists contains articles on many of the points above.

Software and code

Policy information about availability of computer code

Data collection	no software used
Data analysis	R version 4.0.2 was used for data analysis. The script for analysis and additional files are available at: https://osf.io/q4cj5/

For manuscripts utilizing custom algorithms or software that are central to the research but not yet described in published literature, software must be made available to editors and reviewers. We strongly encourage code deposition in a community repository (e.g. GitHub). See the Nature Portfolio guidelines for submitting code & software for further information.

Data

Policy information about availability of data

All manuscripts must include a data availability statement. This statement should provide the following information, where applicable:

- Accession codes, unique identifiers, or web links for publicly available datasets
- A description of any restrictions on data availability
- For clinical datasets or third party data, please ensure that the statement adheres to our policy

All the datasets generated during the current study are included in the supplementary information/source data file. The Li et al. data used in this study are available in the supplemental files of the original article https://doi.org/10.7554/eLife.51701.

Field-specific reporting

Please select the one below that is the best fit for your research. If you are not sure, read the appropriate sections before making your selection.

☒ Life sciences ☐ Behavioural & social sciences ☐ Ecological, evolutionary & environmental sciences

For a reference copy of the document with all sections, see nature.com/documents/nr-reporting-summary-flat.pdf

Life sciences study design

All studies must disclose on these points even when the disclosure is negative.

Sample size	A basic power analysis was performed and determined that for 0.8 power, we should analyse at least 200 for a 10% difference between inheritance rates.
Data exclusions	No data were excluded
Replication	Biological replicates were performed as described in the main article text. As listed in the source data, multiple F1 of the specified genotype/phenotype were used for each cross. No replicates were excluded.
Randomization	Mosquitos of the specified genotype/phenotype were randomly selected for crossing and scoring of the F2 Phenotype.
Blinding	Blinding was not performed as none of the data recorded were subjective.

Reporting for specific materials, systems and methods

We require information from authors about some types of materials, experimental systems and methods used in many studies. Here, indicate whether each material, system or method listed is relevant to your study. If you are not sure if a list item applies to your research, read the appropriate section before selecting a response.

Materials & experimental systems

n/a	Involved in the study	
☒	☐	Antibodies
☒	☐	Eukaryotic cell lines
☒	☐	Palaeontology and archaeology
☐	☒	Animals and other organisms
☒	☐	Human research participants
☒	☐	Clinical data
☒	☐	Dual use research of concern

Methods

n/a	Involved in the study	
☒	☐	ChIP-seq
☒	☐	Flow cytometry
☒	☐	MRI-based neuroimaging

Animals and other organisms

Policy information about studies involving animals; ARRIVE guidelines recommended for reporting animal research

Laboratory animals	Aedes aegypti Liverpool strain (LVP), males and females. LVP has been maintained in culture since 1936
Wild animals	No wild animals were used.
Field-collected samples	No field-collected samples were used.
Ethics oversight	No ethical oversight is required for the insect lines used in this study.

Note that full information on the approval of the study protocol must also be provided in the manuscript.
